# A simulation procedure curriculum to increase pediatric resident exposure to procedures rarely performed in clinical practice

**DOI:** 10.1080/10872981.2019.1611305

**Published:** 2019-05-06

**Authors:** Meera S. Meerkov, Jason B. Fischer, Thomas G. Saba

**Affiliations:** Department of Pediatrics, Michigan Medicine, Ann Arbor, MI, USA

**Keywords:** Pediatrics, procedures, competency, curriculum

## Abstract

**Background**: Pediatrics residents are expected by the Accreditation Council for Graduate Medical Education to competently perform 13 procedures. However, residents are graduating with poor self-perceived competency for these procedures.

**Objective**: We developed a curriculum using simulation training at the beginning of internship and ‘refresher’ workshops throughout the year in order to increase procedure exposure and improve self-perceived competency.

**Design**: Procedural workshops were taught during intern orientation and to all pediatrics residents throughout the academic year. Residents provided a quantitative competency self-assessment before and after each workshop; interns provided an additional self-assessment at the end of the intern year.

**Results**: The curriculum was well-liked and led to more procedural experience. Mean competency self-assessment scores improved immediately after almost every procedure workshop. Mean scores were retained at the end of intern year for most procedures. However, end-of-year mean competency self-assessment and procedural experience on actual patients were similar to interns from a previous year that had not participated in the curriculum.

**Conclusions**: A pre-internship procedure workshop coupled with longitudinal workshops is a feasible way to improve intern exposure to pediatric procedural training. However, it was not sufficient to improve mean competency self-assessments compared to a traditional model of bedside procedural training.

## Introduction

The Accreditation Council for Graduate Medical Education (ACGME) requires that pediatrics residents demonstrate competency in 13 procedures. However, with restrictions on clinical and educational work hours (duty hours) as part of the new Common Program Requirements and integration of advanced practice providers into patient care, pediatrics residents might not be getting adequate opportunities to learn procedural skills [1–3]. As a result, pediatrics residents nationwide are graduating with poor self-perceived competency [2–8]. The current approach to pediatric procedural training is failing to produce pediatricians with procedural competency that meets the expectations of the ACGME and most importantly, our patients and their families. Procedural training at our institution traditionally involved supervised training in vascular access and airway management but does not include a comprehensive approach to address the requirements of the ACGME.

Many attempts to improve procedural competency and confidence in the current educational landscape have been undertaken. Simulation training has been shown to be a safe and effective way to teach procedures and improve both competency and confidence [4]. However, isolated workshops of simulation-based procedural training among pediatrics residents have not consistently led to improved clinical skills on actual patients and knowledge and skills are poorly retained [9–11]. Competency improves with greater experience although the number of procedures needed to achieve competency remains unknown [12].

We hypothesized that a curriculum, based on the ‘learn, see, practice, prove, do, maintain’ framework described by Sawyer et al. [13], would lead to improved and preserved pediatric intern’s competency self-assessment. We aimed to a) quantify the difference in competency self-assessments immediately after and 1 year after the curriculum, b) compare the end-of-year competency self-assessment to a cohort that had not participated in the curriculum, c) assess the impact of this curriculum on the number of procedures performed, and d) measure learner satisfaction.

## Methods

### Procedural workshops

#### Intern orientation workshops

During intern orientation, prior to starting clinical responsibilities, interns were taught eight pediatric procedures in 30-min intervals divided into two half-day workshops (Table 1). We selected procedures that are relatively challenging to perform and for which a simulation model could be developed. Prior to the workshops, participants were provided high-yield procedural summaries describing the procedural steps, indications and contraindications, access to online videos and an online knowledge assessment providing real-time feedback to wrong answers. Completion of knowledge assessments was a pre-requisite to participation but utilization of other resources was not verified. Employing four low-fidelity simulation models at each station, groups of 6–8 participants received a demonstration of the skills and steps, performed deliberate practice and received formative feedback from the expert instructor. Instructors were faculty, fellows or advanced practice providers with procedural expertise. Instructors provided an observed formative assessment of each learner and learners provided a competency self-assessment (as described below). Learners were permitted to move on to the next workshop after the instructor determined that their competency score was above 1.

**Table 1.** Procedure curriculum.

Orientation Workshops	Longitudinal Workshops
Abscess Incision and Drainage*	Abscess Incision and Drainage*
Bag Mask Ventilation*	Bag Mask Ventilation*
Bladder Catheterization	Foreign Body Removal*
Intubation*	Fracture Splinting*
Laceration Repair*	Gastrostomy Tube Management
Lumbar Puncture*	Intraosseous Line Placement
Peripheral IV*/Venipuncture	Intubation*
Umbilical Line Placement*	Laceration Repair*
	NG Tube Insertion
	NJ Tube Bridling
	Peripheral IV Placement*
	Reduction of a Dislocation*
	Tracheostomy Management

*Procedural competency required by the ACGME. NG, nasogastric; NJ, nasojejunal; I&D, incision and drainage; IV, Intravenous.

#### Longitudinal workshops

A voluntary 1-h procedure workshop during protected noon conference was conducted 7 times in the academic year (Table 1). This was available to learners at all levels of training. The procedures taught included many of those required by the ACGME but also procedures frequently performed by residents despite limited training. Two procedures were taught during each 1-h workshop. Each procedure was taught by a single instructor to a group of approximately 15 learners, depending on conference attendance. Teaching resources and strategies, including expert formative feedback, were similar to those used in the intern workshops.

### Competency self-assessments (CSA) score

The CSA score is a 4-point Likert scale based on the entrustable professional activities (EPAs) framework for supervision of activities [14]. The CSA score was defined as: 1 = not competent, 2 = competent with supervision, 3 = competent with indirect supervision, 4 = competent to teach.

Residents were asked to provide a CSA using an electronic survey linked to a Quick Response (QR) code at the beginning of intern year, at the end of intern year and before and after each procedural workshop using the competency scale described above. The 2015–16 class, which did not participate in the curriculum, provided only end-of-year CSA scores.

### Procedural experience

Residents documented all procedures performed in both simulated environments and in the clinical setting. Prior to 2016, residents logged each procedure using an online management system. Starting in 2016, procedures were logged in real-time at the bedside in a database linked to QR code on the residents’ smartphones, providing an opportunity for performance-based feedback from supervisors.

### Learner satisfaction assessments

At the completion of every workshop in the intern and longitudinal curriculum, learners were asked to complete an online, anonymous survey eliciting their level of satisfaction with the workshops. This survey aimed to assess the resident’s overall impression of each simulation station during the workshop, quality of the instructors’ education techniques, utility of the simulation models, and didactic educational content.

The project was reviewed by The University of Michigan Institutional Review Board and was found to be exempt.

### Statistical analysis

The means CSA scores and the mean procedural numbers were compared using unpaired *t* tests. Given the paucity of rigorous data on which to base this study and the fixed sample size, a power calculation was not appropriate for this study. Comparisons were considered statistically significant if p < 0.05. Data analysis was accomplished using GraphPad Prism version 7.00 for Windows, GraphPad Software, La Jolla, California USA, www.graphpad.com.

## Results

All 24 incoming pediatrics interns in categorical Pediatrics, Pediatric Neurology and Pediatric Genetics programs participated in the intern orientation in 2016; eight additional Medicine-Pediatrics interns participated in 2017. Response rates to pre-curriculum CSA, post-curriculum CSA and end-of-year CSAs were 100%, 100% and 75% for the 2016–17 class and 100%, 91% and 53% for the 2017–18 class, respectively. Fifty-eight percent of the 2015–16 class completed the end-of-year CSA. All 96 Pediatric and Medicine-Pediatric residents were invited to participate in the longitudinal procedural curriculum labs in the 2016–17 and 2017–18 academic years. Approximately 20–30 residents participated in each of the longitudinal curriculum workshops and there was a relatively even distribution of participation among first-, second- and third-year Pediatrics residents. Workshops were intentionally spaced out throughout the year in order to assure a diverse distribution of learners at each workshop.

**Figure 1.**
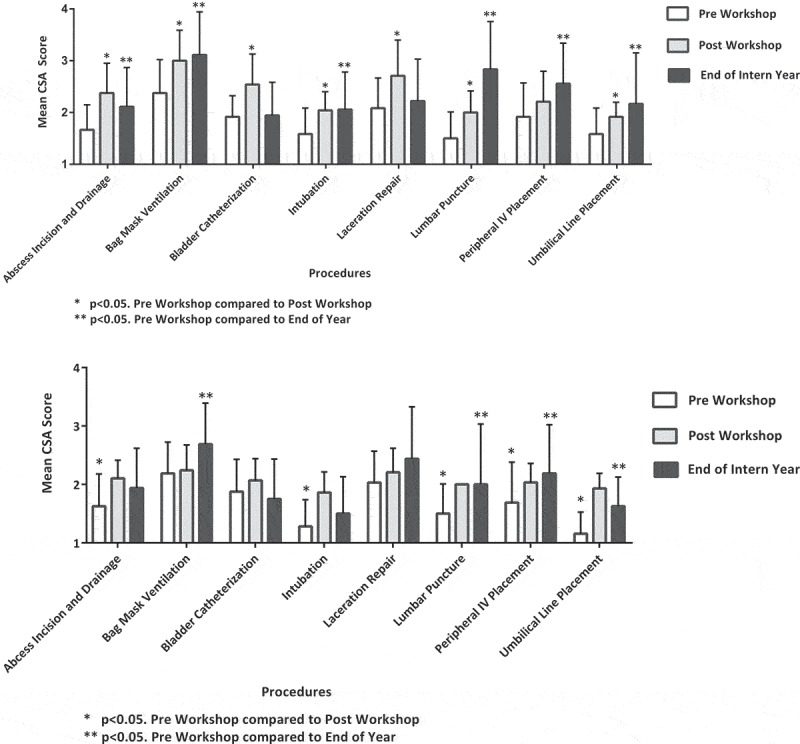
(a) Mean competency self-assessment (CSA) score by procedure – 2016. (b) Competency self-assessment (CSA) score by procedure – 2017.

Immediately after the 2016 intern curriculum, the mean CSA scores of interns improved for all procedures taught except peripheral IV placement (PIV) (Figure 1(a)). After the 2017 intern curriculum, the mean CSA scores improved for all procedures except bag mask ventilation (BMV), bladder catheterization and laceration repair.

**Figure 2.**
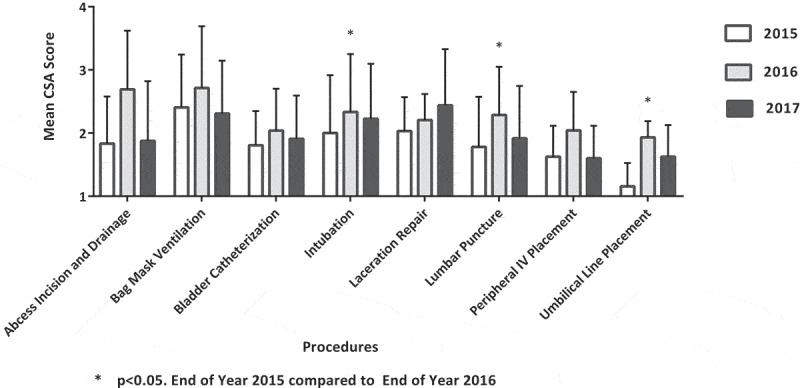
End of intern year mean competency self-assessment (CSA) score – 2015, 2016, 2017.

CSA scores were measured at the end of intern year in order to assess retention of skills acquired during orientation. End-of-year mean CSA scores were greater than the pre-curriculum levels for all procedures except for bladder catheterization and laceration repair for the 2016–17 cohort (Figure 1(a)). For the 2017–18 cohort, end-of-year mean CSA scores were greater than the pre-curriculum scores for all procedures except for bladder catheterization, laceration repair, abscess incision and drainage (I&D) and intubation (Figure 1(b)).

At the end of the intern year, the 2016–17 cohort mean CSA scores for lumbar puncture (LP), neonatal intubation and umbilical line placement (ULP) were greater than that of the 2015–16 cohort, which had not participated in the curriculum. Otherwise, the end-of-year mean CSA scores of the 2016–17 and 2017–18 cohorts were similar to the mean CSA scores of the 2015–16 cohort (Figure 2).

The number of procedures performed overall increased by 53% in the 2016–17 class and by 36% in the 2017–18 class, compared to the 2015–16 class. Importantly, every intern had exposure, via simulation, to procedures infrequently performed at our institution by pediatric residents such as abscess I&D and bladder catheterization. The number of procedures performed in clinical, non-simulation settings, however, was similar among all three intern years (Table 2). Interns within each cohort performed approximately the same number of procedures. The most commonly performed procedures on patients in all three intern years were BMV, LP and PIV (Table 2). The end-of-year mean CSA scores were greatest for BMV, LP and PIV in the 2016–17 year and BMV, laceration repair and PIV in the 2017–18 year.

**Table 2.** Number of procedures performed by interns.

	Total procedures			Procedures performed in clinical setting	
**Procedure**	**2015**	**2016**	**2017**	**2016**	**2017**
Abscess Incision and Drainage	10	39	38	7	7
Bag Mask Ventilation	78	78	78	46	49
Bladder Catheterization	5	26	28	1	3
Intubation	39	70	53	38	25
Laceration Repair	29	42	49	18	24
Lumbar Puncture	46	79	57	53	32
Peripheral IV placement	67	100	80	73	65
Reduction of Simple Dislocation	7	17	11	14	7
Removal of Foreign Body	5	11	12	4	9
Splinting of a Fracture	6	12	8	9	2
Umbilical Catheter Placement	37	48	47	22	18
Venipuncture	24	17	19	14	11
Total	353	539	480	299	252

Following the longitudinal curriculum workshops, mean CSA scores for all residents improved for 8 of the 13 procedures taught. CSA scores did not improve for the following procedures: BMV, reduction of a dislocation, laceration repair, LP and foreign body removal.

This procedural curriculum was generally well-liked by residents. At least 93% of interns reported a positive experience at each procedure workshop and satisfaction with the models used except for the bladder catheterization station. At this station, only 64% agreed that the videos, procedural summaries and knowledge assessments were helpful. Ninety-eight percent of those that attended one of the longitudinal procedure labs agreed or strongly agreed that the session was a positive experience and 95% agreed or strongly agreed that the simulation models were helpful. Residents provided comments describing the poor representation of the model used to teach foreign body removal.

## Discussion

In this study, we developed a pediatric procedural curriculum employing a simulation-based “crash course” for interns and monthly ‘refresher’ workshops throughout the year. The curriculum led to a significant increase in procedure exposure, particularly among procedures rarely performed by pediatric residents at our institution in a clinical setting. Mean CSA scores improved as a result of the simulation-based workshops for most, but not all, of the procedures taught and was mostly retained by the end of the year. However, end-of-year mean CSA scores were no better than the mean CSA scores of the intern class that had not participated in the curriculum.

Mean CSA scores improved following the intern orientation procedural simulation workshop for most, but not all procedures taught to the 2016–17 and 2017–18 intern cohorts. Although the curriculum and models used received positive feedback, the quality of the simulation manikins/devices may have contributed to the variability in CSA score improvement. For example, the model used to teach abscess I&D, which led to a significant increase in mean CSA score, was designed uniquely for this workshop using a combination of lotion and make-up to represent a pediatric skin abscess. On the other hand, the models used to teach foreign body removal and reduction of simple dislocations in the longitudinal workshops were simple, low-fidelity body parts not designed for these particular procedures. Following the sessions that utilized these models, mean CSA scores did not improve. Adopting a successful curriculum for pediatrics residents that will result in improved competency and confidence, therefore, will require careful design of didactic and simulation teaching materials. In this study, better quality teaching materials for some workshops might have led to a greater impact.

Lack of mean CSA score improvement for some procedures might have been due to relatively strong mean CSA score prior to the workshops (Figure 1(a,b)). Mean CSA scores for BMV and laceration repair workshops did not improve in the longitudinal curriculum in 2016–17 possibly because pre-workshop mean CSA scores levels were already relatively high (2.91; SEM± 0.13 and 2.61; SEM± 0.16, respectively). It is not surprising that a simulation workshop didn’t help residents who felt almost ready to perform these procedures independently even prior to this educational experience.

Mean CSA scores were retained at the end of the academic year for the majority of procedures taught during the intern procedural curriculum for the 2016–17 and 2017–18 classes. Interestingly, year-end mean CSA scores for the 2016–17 and 2017–18 intern classes compared to the 2015–16 class were similar for most procedures. Of note, intern procedural experience on actual patients was also similar between all three intern classes and there were no significant changes to the overall intern clinical or curricular structure other than the implementation of the simulated workshops starting with the 2016–17 class. This could lead to the assumption that end-of-year CSA scores were largely a result of actual clinical experience, as the interns who did not have the opportunity to participate in the procedural curriculum had similar end-of-year CSA scores. A study by Augustine et al. identified a positive correlation between the number of procedures performed and procedural confidence at the time of a simulated workshop. Similarly, in the present study, the procedures for which year-end mean CSA scores were highest (BMV, LP, PIV) were the procedures most frequently performed on actual patients. Whereas the Augustine study elicited procedures performed prior to the simulated training, our study looked at procedures performed after the simulated training [4]. Procedural experience on actual patients has been shown to be critically important to gaining competence, but resident exposure is insufficient [12]. The relative impact of simulation training and actual procedural experience on CSA would have to be evaluated with more direct investigation.

Our study had limitations. CSA is not a true measure of procedural competency [15–17]. A structured, performance-based competency assessment was beyond the scope of this study. Instead, our study was designed to describe a feasible and practical way of implementing procedural training and increasing procedure exposure in pediatric residency training programs. We would argue that, although self-assessment is an unreliable measure of true procedural competency at one point in time, identifying improvement in CSA over time likely reflects a true difference in procedural competency and a valuable measurement of the success of the curriculum. The CSA, however, is not designed to accurately quantify the size of the impact. Further studies are needed in order to determine whether this training model leads to procedural competency using more objective measures. Lastly, our study did not compare mean pre- and post-CSA scores in a paired fashion, although nearly all learners completed the pre- and post-workshop CSAs.

In conclusion, a mandatory procedural workshop is a feasible and practical way to increase exposure to procedural experience among pediatrics interns. Interns gained exposure to procedures that, prior to this curriculum, some interns had never performed during residency. Studying the outcomes of this curriculum helped to identify the important role of bedside procedural experience with actual patients. In order to optimize procedural education in residency, application of the Sawyer ‘learn, see, practice, prove, do, maintain’ model should be paired with greater opportunities for deliberate procedural experience in a clinical setting in order to improve confidence and competence of pediatric residents at the time of graduation. Further studies are needed to determine the applicability of this model to medical learners at different educational levels and specialties.
